# The San Francisco Medical Press

**Published:** 1862-03

**Authors:** 


*The San Francisco Medical Press* shows an enterprise and liberality worthy of especial mention. With the view of stimulating the profession of the Pacific Coast to subscribe for other medical journals, it makes the following proposition:

"We will send the *Press* to any person for $1.00 per annum, who is, or shall become, a subscriber to any one of the following exchanges; to any one who shall become a subscriber to two, fifty cents per annum; and *free of charge to all who are, or shall become subscribers to three:*"

Pacific Medical and Surgical Journal,
London Lancet,
Summary of Medical Science,
American Journal of the Medical Sciences,
American Journal of Pharmacy,
American Medical Monthly.
American Medical Times,
Berkshire Medical Journal,
Boston Medical and Surgical Journal,
British & Foreign Medico-Chirurgical Review,
Buffalo Medical and Surgical Journal and Reporter,
Cincinnati Lancet and Observer,
Cincinnati Medical and Surgical News,
Chicago Medical Examiner,
Chicago Medical Journal,
Gazette des Hospitaux,
Journal of Materia Medica,
Medical News and Library,
Medical and Surgical Reporter,
N. A. Medico-Chirurgical Review,
St. Louis Medical & Surgical Journal.

Such devotion to the profession, and effort for the advancement of Medical literature; such unselfish, whole-souled, liberal-hearted effort, is worth more, and will accomplish more for the real good of the profession than we have language to express. We have no need to say, after what has been quoted, that the San Francisco Medical Press is one of the most valuable medical journals in the English language; every physician would know as much without further comment. It is edited by E. S. Cooper, A. M. M. D., Professor of Anatomy and Surgery in the Medical Department of the University of the Pacific.

